# Biocompatibility Assessment of Novel Collagen-Sericin Scaffolds Improved with Hyaluronic Acid and Chondroitin Sulfate for Cartilage Regeneration

**DOI:** 10.1155/2013/598056

**Published:** 2013-11-07

**Authors:** Sorina Dinescu, Bianca Gălăţeanu, Mădălina Albu, Adriana Lungu, Eugen Radu, Anca Hermenean, Marieta Costache

**Affiliations:** ^1^Department of Biochemistry and Molecular Biology, University of Bucharest, 91-95 Splaiul Independentei, 050095 Bucharest, Romania; ^2^Collagen Department, Leather and Footwear Research Institute, 93 Ion Minulescu, 031215 Bucharest, Romania; ^3^Advanced Polymer Materials Group, Department of Bioresources and Polymer Science, University Politehnica of Bucharest, 149 Calea Victoriei, 010072 Bucharest, Romania; ^4^Molecular Biology and Pathology Research Lab “Molimagex”, University Hospital Bucharest, 169 Splaiul Independentei, 050098 Bucharest, Romania; ^5^Department of Histology, Faculty of Medicine, Pharmacy and Dentistry, Vasile Goldis Western University of Arad, 1 Feleacului, 310396 Arad, Romania; ^6^Department of Experimental and Applied Biology, Institute of Life Sciences, Vasile Goldis Western University of Arad, 86 Rebreanu, 310414 Arad, Romania

## Abstract

Cartilage tissue engineering (CTE) applications are focused towards the use of implantable biohybrids consisting of biodegradable scaffolds combined with *in vitro* cultured cells. Hyaluronic acid (HA) and chondroitin sulfate (CS) were identified as the most potent prochondrogenic factors used to design new biomaterials for CTE, while human adipose-derived stem cells (ASCs) were proved to display high chondrogenic potential. In this context, our aim was not only to build novel 3D porous scaffolds based on natural compounds but also to evaluate their *in vitro* biological performances. Therefore, for prospective CTE, collagen-sericin (Coll-SS) scaffolds improved with HA (5% or 10%) and CS (5% or 10%) were used as temporary physical supports for ASCs and were analyzed in terms of structural, thermal, morphological, and swelling properties and cytotoxic potential. To complete biocompatibility data, ASCs viability and proliferation potential were also assessed. Our studies revealed that Coll-SS hydrogels improved with 10% HA and 5% CS displayed the best biological performances in terms of cell viability, proliferation, morphology, and distribution. Thus, further work will address a novel 3D system including both HA 10% and CS 5% glycoproteins, which will probably be exposed to prochondrogenic conditions in order to assess its potential use in CTE applications.

## 1. Introduction

Regenerative medicine is a multidisciplinary field of research which involves the use of biomaterials, growth factors, and stem cells in order to repair, replace, or regenerate tissues and organs damaged by injury or disease [1]. Consequently, it has evolved tremendously in the last decade together with the advances in the biotechnological field. Currently, tissue engineering applications are focused towards the use of implantable biohybrids consisting of biodegradable scaffolds combined with *in vitro* cultured cells, as a regeneration strategy. 

Cartilage tissue engineering (CTE) has been increasingly explored in the recent years [2, 3], as cartilage damages cause disabilities to more than 200 million of middle age and older people from all over the world [4]. Due to the cartilaginous tissue's particularities, CTE requires crucial combinations of cells and biomaterials [5]. The complexity and the specificity of the cartilage reside in its aneural, avascular, and alymphatic nature [6]. More specifically, the adult cartilage tissue has a limited self-repair potential “due to the sparse distribution of highly differentiated, nondividing chondrocytes, slow matrix turnover, low supply of progenitor cells, and lack of vascular supply” [7]. Consequently, the task assigned to tissue engineering applications is difficult as there were no sufficient successful approaches to reproducibly regenerate functional cartilage up to date. In this context, cartilage regeneration represents one of the most difficult challenges in the field of tissue engineering and clinical applications.

Novel scaffolds which facilitate the differentiation of stem cells into cartilaginous phenotype concomitant with their assembly into 3D tissue [3] play an important role as extracellular matrix (ECM) [8]. So far, a wide range of natural and synthetic polymers were investigated as scaffolds for CTE [9]. Encouraging results in cartilage reconstruction applications were obtained using collagen-based matrices associated with chondrocytes [10] or MSCs [11]. Collagen-based scaffolds are widely used in tissue engineering and previous studies have shown successful results in the development of novel 3D systems designed for adipose tissue reconstruction using collagen biomatrices improved with sericin and preseeded with ASCs [12]. Silk sericin (SS), a natural macromolecular protein surrounding *Bombyx mori* silk fibers [13], was shown to be responsible for the proliferation and attachment of several mammalian cell lines [14–16] as well as for the activation of collagen production, both *in vitro* and *in vivo* [17–19]. Based on these properties, SS was included in the composition of our scaffolds in the view of cartilage reconstruction.

To successfully mimic the cartilage tissue's environment, the fundamental structure of the designed biomaterial should be a tridimensional system [20]. To date, the following potential scaffolds for CTE applications were developed: hybrid poly-(lactic-co-glycolic acid)-gelatin/chondroitin/hyaluronan [21], gelatin-chondroitin-hyaluronan tri-copolymer [8], chitosan-based hyaluronic acid hybrid biomaterial [20], chondroitin-6-sulfate/dermatan sulfate/chitosan [22], injectable chitosan-hyaluronic acid [23], enzymaticallycross-linked injectable hydrogel-based biomimetic dextran-hyaluronic acid [24], poly (*γ*-glutamic acid)-graft-chondroitin sulfate/polycaprolactone [25], hyaluronic acid-gelatin-chondroitin sulfate [26], and chitosan-hyaluronic acid hydrogels [4].

Among the most beneficial prochondrogenic factors used in the design of new biomaterials for CTE are hyaluronic acid (HA) and chondroitin sulfate (CS). HA is a natural molecule component of the ECM from many tissues, including cartilage, with multiple physical and biological functions. HA plays a vital role in the development of cartilage, the maintenance of the synovial fluid, and the regeneration of tendons [27]. HA has been extensively investigated for tissue engineering applications due to its biocompatibility, biodegradability, and readily modified chemical structure. HA was embedded in multiple scaffold compositions, where it increased the ability of chondrocytes to synthesize ECM-specific biomolecules *in vitro *and *in vivo* [28]. The chondroprotective effects of hyaluronic acid and the potential to stimulate the production of tissue inhibitors of matrix metalloproteinases (TIMP-1) in chondrocytes inhibit cartilage degradation [29]. Articular chondrocytes cultured in the presence of HA had a significantly greater rate of proliferation, and ECM production, compared to chondrocytes cultured in the absence of HA [30]. 

CS is one of the natural glycosaminoglycans (GAG) found in the structure of the aggrecan molecule of the cartilage. Among other properties, CS is responsible for the water retention of cartilage, due to the negative charge ensured by its structure [31]. CS is involved in the intracellular signaling, cell recognition and connection of ECM components to cell-surface glycoproteins [32, 33] and collagen. CS has a number of useful biological properties for cartilage engineering including anti-inflammatory activity, would healing, the ability to inhibit the enzymes responsible for cartilage degradation, and a biological activity at the cellular level that helps restore arthritic joint function [34]. 

Regarding the cellular component of the implantable biohybrids, stem cells are ideal candidates for regenerative medicine due to their ability to commit to multiple cell lineages and to self-renew [35, 36]. Previous experience with tissue reconstruction showed that stem cells used in these applications should meet the following criteria [35]: (i) abundance of cells; (ii) minimally invasive procedure with minimal morbidity harvest; (iii) differentiation potential along multiple cell lineages in a controllable and reproducible manner; (iv) safe transplantation to either an autologous or allogeneic host; and (v) possibility of isolation in accordance with the current Good Manufacturing Practice guidelines.

Several sources of stem cells are likely to meet these requirements, yet human adipose-derived stem cells (ASCs) have multiple benefits [37]. Subcutaneous adipose tissue is accessible and thus ASCs can be harvested in large quantities with minimal risk. In addition, adipose tissue yields manifold greater numbers of mesenchymal stem cells (MSCs) compared to bone marrow [37]. Once implanted at the injury site, the differentiating ASCs not only generate a filling tissue, but, due to their secretory profile, they are able to modulate the recruitment of the endogenous stem cells and to promote their differentiation towards the required lineage pathway [38]. Consequently, ASCs secrete almost all of the growth factors involved in normal wound healing [13, 39].

ASCs can be reproducibly isolated from liposuction aspirates through a procedure involving collagenase digestion, differential centrifugation, and expansion in culture [40]. Undifferentiated ASCs express a distinct immunophenotype detectable by flow cytometry (CD29^+^, CD44^+^, CD73^+^, CD90^+^, CD105^+^, CD166^+^ and CD14^−^, CD31^−^, CD45^−^) [12, 41] and produce additional adipocyte-specific proteins upon induction [40]. 

ASCs have the potential to differentiate into bone, cartilage and muscle as well as adipose and neural tissue [41–47]. This ability to differentiate towards different mesenchymal lineages has stimulated interest in their clinical use. The chondrogenic potential of ASCs has been validated *in vitro *using a variety of culture systems, growth factors, and differentiation culture conditions. It was reported that after the exposure to conditioned chondrogenic media, ASCs commit and differentiate towards chondrogenic lineage, expressing higher levels of chondrocyte-associated genes such as type II collagen and aggrecan [48]. 

In this context, our aim was not only to build novel 3D porous scaffolds based on sericin and collagen, improved with prochondrogenic factors such as chondroitin sulfate or hyaluronic acid for CTE, but also to evaluate their *in vitro *biological performance. Therefore, the biocompatibility of collagen-sericin (Coll-SS) scaffolds improved with HA (5% or 10%) and CS (5% or 10%) was compared to a reference hydrogel (Coll-SS) in order to identify an appropriate environment for prospective CTE. These temporary physical supports for ASCs were analyzed in terms of structural (FTIR spectroscopy), thermal (DSC), morphological (SEM), and swelling properties and cytotoxic potential (LDH). Considering that these hydrogels were designed for prospective CTE applications and that ASCs display high chondrogenic potential, this particular type of adult stem cells was used for scaffold biocompatibility evaluation.

## 2. Materials and Methods

### 2.1. Cell Culture Model

Human adipose-derived stem cells (ASCs) provided by Invitrogen (Life Technologies, Foster City, CA, USA) were used for this study. Cells were isolated from human lipoaspirate tissue, then they were expanded for one passage in MesenPRO RS Medium (Invitrogen, Life Technologies, Foster City, CA, USA), a low (2%) serum concentration medium that reduces ASCs doubling times and finally they were cryopreserved from primary cultures. According to the manufacturer, each lot of ASCs originates from a single donor of human subcutaneous adipose tissue and the cells can be expanded for 4-5 passages before losing their ability to grow or differentiate into all potential phenotypes, including adipocytes, osteoblasts, and chondrocytes. These ASCs express the following cell-surface markers profile: CD29^+^, CD44^+^, CD73^+^, CD90^+^, CD105^+^, CD166^+^ and CD14^−^, CD31^−^, CD45^−^, and Lin1^−^.

### 2.2. Subcultivation

The culture was propagated for two passages and split 1 : 4 after achieving 75–80% confluence in order to achieve the cell number required for 3D cultures assessment. Therefore, cells plated in T75 culture flasks (Nunc, Thermo Scientific, Waltham, MA, USA) were incubated in MesenPRO RS Medium, at 37°C in a humidified atmosphere of 5% CO_2_ and 95% air, with growth media changed every 48 h. 

Cell morphology was observed by phase contrast microscopy (Nikon Eclipse TS 100, Nikon Instruments Europe, Amsterdam, Netherlands) every day.

### 2.3. Preparation of 3D Coll-SS-Based Porous Biomatrices

Type I collagen gel (Coll) with an initial concentration of 2.11% and pH 2.8 was extracted from calf hide using the technology previously described [49]. SS was purchased from Sigma-Aldrich (Shinagawa-Ku, Tokyo, Japan), chondroitin sulfate sodium salt from shark cartilage (CS), and hyaluronic acid potassium salt, from Human Umbilical Cord (HA) from Sigma-Aldrich Chemie GmbH (Steinheim, Germany) and glutaraldehyde (GA) was received from Merck (Darmstadt, Germany). 

The cartilage scaffolds based on collagen, sericin, and glycosaminoglycans were prepared in a similar manner as previously described by Dinescu et al. [50] and Lungu et al. [51, 52]. The reference hydrogel at pH 7.4 consists of 1.2% collagen solution and 40% SS reported to collagen dry substance. The cross-linking was performed using 0.5% GA (reported to dry collagen). Loading of 5 and 10% HA or CS, respectively, to the previously described Coll-SS composition, considered as a reference sample, resulted in several mixtures with the following ratios: Coll : SS : HA = 100 : 40 : 10, Coll : SS : HA = 100 : 40 : 5, Coll : SS : CS = 100 : 40 : 10, and Coll : SS : CS = 100 : 40 : 5. Further on, a freeze-drying process was performed according to [51] in order to obtain spongious scaffolds.

The novelty of these scaffolds resides not only in the original combination of collagen, chondroitin sulfate or hyaluronic acid with sericin but also from the 3D porous nature of the scaffolds.

### 2.4. Achievement of 3D Cultures

ASCs in the 3rd passage were seeded on top of Coll-SS, Coll-SS : HA 10%, Coll-SS : HA 5%, Coll-SS : CS 10% and Coll-SS : CS 5% biomatrices at an initial density of 2 × 10^5^ cells/cm^2^. The cell suspension was allowed to diffuse through the hydrogels in order for the cells to adhere to the biomaterial. After 1 hour, the resulting 3D bioconstructs were incubated in standard conditions of cultivation in MesenPRO RS Medium. In our experiments, we defined as bioconstructs the porous 3D hybrids resulting after Coll-SS-based hydrogels were put in contact with ASCs.

For further simplicity, the following abridgements will be introduced to designate the studied 3D scaffolds: Coll-SS = control; Coll-SS : HA 10% = sample A; Coll-SS : HA 5% = sample B; Coll-SS : CS 10% = sample C; Coll-SS : and CS 5% = sample D. 

### 2.5. Scaffolds Characterization

#### 2.5.1. Scaffolds Physicochemical and Morphological Characterization

The CTE-designed scaffolds were characterized by Fourier transform infrared (FTIR), thermal analysis, scanning electron microscopy (SEM) and water uptake. 

*FTIR* spectra were registered on a VERTEX 70 BRUCKER FTIR spectrometer equipped with an attenuated total reflectance (ATR) accessory. All FTIR measurements were performed in the ATR-FTIR cell on Ge crystal, at room temperature. The FTIR spectra were recorded using 32 scans in 600–4000 cm^−1^ wavenumber region. 

Thermal properties of the obtained matrices were determined by differential thermal calorimetry (DSC) using a Netzsch DSC 204 F1 Phoenix equipment. Samples of about 2 mg were heated from 20 to 300°C under a constant nitrogen flow rate (20 mL/min). A heating rate of 10°C/min was applied. 

Scanning electron microscopy (SEM) was used to determine the morphological structure of the scaffolds. The analysis was performed using a QUANTA 200 SEM device. 

*The water absorption* was determined gravimetrically, as previously described [53].

#### 2.5.2. Biocompatibility Assessment of 3D Coll-SS-Based Biomaterials

Biocompatibility was evaluated in terms of the viability and the proliferative activity of ASCs in contact with the control and samples A, B, C, and D using qualitative Live/Dead Assay and quantitative MTT test. The cytotoxic potential of the biomaterials on ASCs was evaluated by spectrophotometric quantification of the LDH released in culture medium. 

*Live/dead fluorescence microscopy assay was performed* to evaluate ASCs viability and proliferation within the 3D culture systems, using Live/Dead kit (Invitrogen, Life Technologies, Foster City, CA, USA). This fluorescence-based kit combines calcein AM and ethidium bromide to yield two-color discrimination of the population of live cells from the dead-cell population. Calcein AM is a non-fluorescent and permeable reagent, which is converted by the intracellular esterases into the intensely green fluorescent calcein (ex/em: ~495 nm/~635 nm). Ethidium bromide enters the cells with damaged membrane, producing a bright red fluorescence when binding to nucleic acids (ex/em: ~495 nm/~635 nm). 

Briefly, at 2, 4, and 7 days after seeding, the control, A, B, C, and D bioconstructs were incubated with a staining solution prepared according to manufacturer's instructions for 15 minutes at dark. Next, the stained 3D cultures were analyzed by fluorescence microscopy using an Olympus IX71 inverted microscope and images were captured with Cell F Imaging Software (Olympus, Hamburg, Germany, 2008). 

In addition, confocal 3D images were acquired with a Carl Zeiss LSM710 laser-scanning confocal microscopy system using Zeiss 20x 0.5NA objective. Carl Zeiss Zen 2010 software version 6.0 was used for image acquisition and analysis. The 488 and 543 nm laser lines were used for excitation and fluorescence emission was detected at 520–550 nm for calcein and 600–680 nm for ethidium bromide. The confocal aperture used corresponded to a backprojected size of 1 Airy unit. Images were acquired as z-stacks using depth brightness correction, and a maximal projection algorithm was used for 3D reconstruction.

A combination approach, consisting of MTT and lactate dehydrogenase (LDH) assays, was used to provide information about cell viability and possible cytotoxic effects of the analyzed materials.

*The MTT assay* allows the evaluation of cell survival by reduction of a tetrazolium salt solution—MTT (3-(4,5-dimethylthiazolyl-2)-2,5-diphenyltetrazolium bromide) to insoluble purple formazan crystals by all living, metabolically active cells. The amount of formazans produced can be determined after solubilization in isopropanol by spectrophotometric quantification at 550 nm. The concentration of the formazan solution is proportional to the amount of the living cells in the culture. Absorbance values, that are lower than those displayed by control cells, indicate a reduction in the cell metabolic activity and viability. Conversely, a higher absorbance rate indicates an increase in cell viability. 

ASCs capacity to proliferate into Coll-SS, Coll-SS : HA 10%, Coll-SS : HA 5%, Coll-SS : CS 10%, and Coll-SS : CS 5% biomatrices was quantitatively determined using MTT spectrophotometric assay at 2, 4, and 7 days afte seeding. In this context, all cell-scaffold bioconstructs studied were incubated in 1 mg/mL MTT (thiazolyl blue tetrazolium bromide) solution (Sigma Aldrich Co., Steinheim, Germany) and after 4 h the formazan crystals were solubilized in isopropanol for 1 h. The absorbance of the resulting solution was measured by spectrophotometry at 550 nm (Appliskan Thermo Scientific, Waltham, MA, USA).

*The LDH Assay* is based on the quantification of the cytosolic lactate dehydrogenase enzyme released in the culture medium by the cells with damaged membrane. 

The environmental cytotoxic potential of the Coll-SS, Coll-SS : HA 10%, Coll-SS : HA 5%, Coll-SS : CS 10% and Coll-SS : CS 5% materials on the ASCs was evaluated using “*In vitro* toxicology assay kit lactate dehydrogenase based” (Sigma Aldrich Co, Steinheim, Germany) according to the manufacturer's protocol. Briefly, the culture media were harvested at 2, 4, and 7 days after seeding and they were mixed with the solutions provided in the kit. After 20 minutes of incubation at room temperature and darkness, the reaction was stopped with 1N hydrochloric acid (HCl). The LDH concentration was determined by measuring the optic density of the resulting solutions at 490 nm (Appliskan Thermo Scientific, Waltham, MA, USA).

The spectrophotometrical data were statistically analyzed using GraphPad Prism 3.03 Software, one-way ANOVA, Bonferroni test. Data are presented as the average of three replicates (mean ± standard deviation).

## 3. Results and Discussion

### 3.1. Scaffolds Physical Characterization

#### 3.1.1. Fourier Transform Infrared (FTIR) Spectra

Freeze drying of collagen-sericin-glycosaminoglycan gel compositions led to porous 3D sponges, which resembled the ECM of cartilage tissue. The properties of these spongious polymeric samples were evaluated by various tests. 

**Figure 1:**
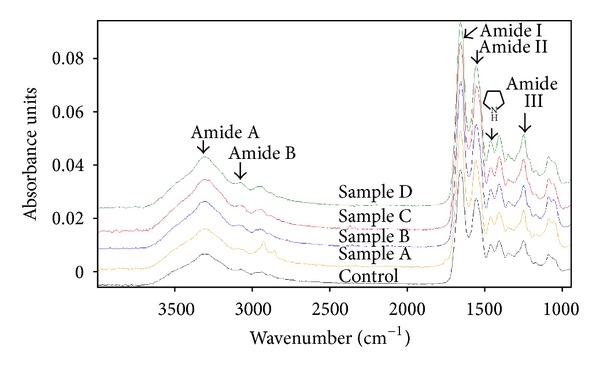
FTIR spectra of Coll-SS-based scaffolds.

FTIR spectra of Coll-SS, Coll-SS : HA, and Coll-SS : CS (with 5% glycosaminoglycan) are shown in Figure 1.

The spectra of Coll-SS scaffolds are characterized by typical protein absorption bands. As revealed by FTIR analysis, the amide A band appeared at 3296 cm^−1^, amide B at 3075 cm^−1^, amide I at 1645 cm^−1^, amide II at 1545 cm^−1^ and amide III at 1241 cm^−1^ in IR spectra. The band of 1452 cm^−1^ is typical for the pyrolidonic ring of hydroxyproline and gives information about the denaturation degree of the collagen triple helix. The ratio between intensity of Amide III and 1452 cm^−1^ was higher than the one for all the studied samples, which indicated that no alterations or significant changes took place. The spectra of the samples are very similarls due to the small content of HA or CS which do not influence the FT-IR spectroscopy.

#### 3.1.2. Thermal Aanalysis

The differential thermal calorimetry (DSC) thermograms showed thermal denaturation caused by the breaking of hydrogen bonds which stabilize the collagen native helical structure. The results of thermal analysis are presented in Table 1.

Collagen is denaturated when heating between 56 and 66°C. The differences in the denaturation temperatures between samples are most probably due to the glycosaminoglycans interaction with Coll-SS and also due to their different ratio. The DSC results showed a strong increase of denaturation temperature for samples containing 10% HA and a decrease for the sample with CS. The melting temperatures showed insignificant differences between samples, and the most ordered structure was noticed for the sample A. Thermooxidation temperatures for all the samples were found to be at about 309°C.

#### 3.1.3. Scanning Electron Microscopy (SEM)

**Figure 2:**
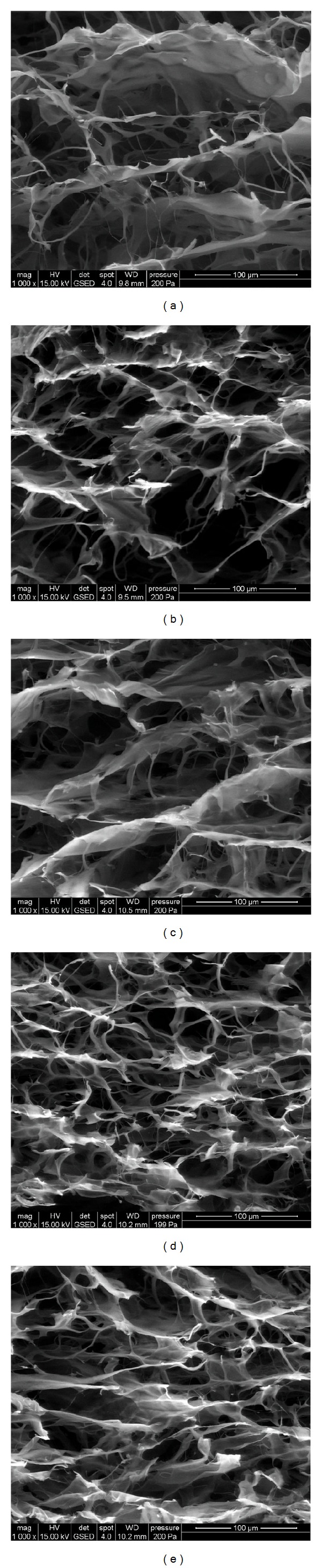
SEM images of (a) Coll-SS, (b) sample A, (c) sample B, (d) sample C and, (e) sample D.

Similarities between structures could also be seen in the SEM images presented in Figure 2.

The porous structures with inner pores interconnected by collagen fibrils were visible in all studied samples, as shown in Figure 2. Pore sizes varied between 20 and 150 *μ*m, being larger for Coll-SS samples which contain HA and smaller for samples which contain CS. When comparing samples A and B, we noticed that a higher amount of HA (10%) induced much homogeneous porosity and larger pore sizes. These results are in accordance with water uptake assessment. We could anticipate that such a structure could further allow a higher mobility of cells inside scaffolds' internal structure during cell proliferation.

#### 3.1.4. Swelling Properties

**Figure 3:**
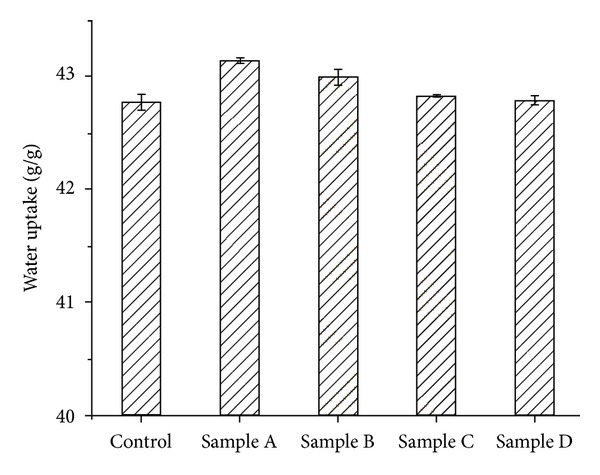
Water uptake of Coll-SS-based scaffolds after 1 h.

The morphology of the samples was also investigated by water uptake and the results are presented in Figure 3. 

According to our results, the scaffolds became stable in terms of water uptake after 1 h of immersion. Although the scaffolds showed similar values of water uptake with variations between 42.80 and 43.15 g/g, sample A and sample B displayed higher hydrophilic character, probably due to their HA content.

### 3.2. *In Vitro* Biocompatibility Assessment

#### 3.2.1. Live/Dead Fluorescence Microscopy Assay

Cell behavior in terms of viability, proliferation, morphology, and distribution, was qualitatively investigated after 2, 4, and 7 days of culture in standard conditions by fluorescence and confocal microscopy, based on the simultaneous staining of live (green labeled) and dead (red labeled) cells.

**Figure 4:**
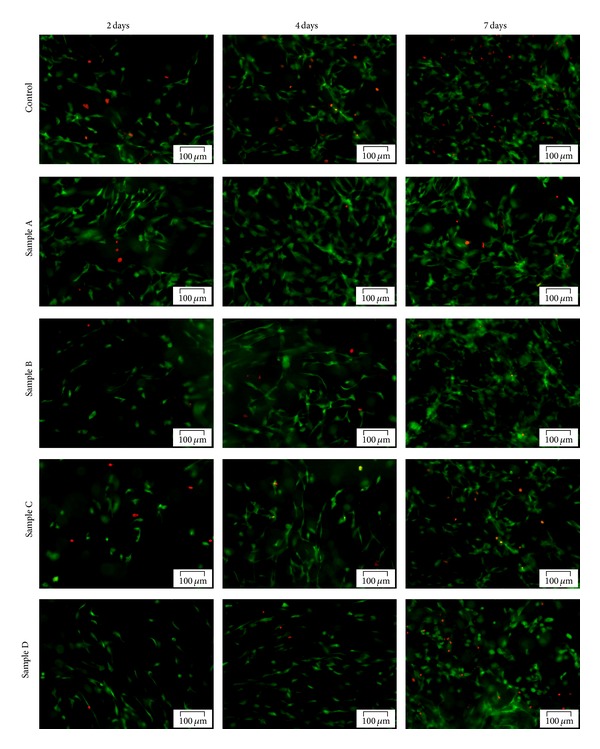
Fluorescence microscopy assessment of living (green-labeled) and dead (red-labeled) ASCs at 2, 4, and 7 days post-seeding on samples A, B, C, D and the control.

High cellular viability was revealed by Live/Dead assay (Figure 4) on the surface of all studied compositions, as well as on the control scaffold during one week of culture. This observation was confirmed by the low amount of dead cells found in all cell-material systems. However, cell density on the surface of sample A was visibly higher at 2 days after seeding than on all other samples and the control, suggesting a higher proliferation rate of ASCs in the presence of 10% HA. Moreover, the lowest cell density at 2 days of culture was observed in samples B and C. These observations were consistent with ASCs density at 4 days after seeding, when the highest proliferation rates were registered in the presence of 10% HA and 5% CS. After 7 days of culture, proliferation was observed in all samples, particularly in samples A and D.

Additionally, cell distribution on the surface of the hydrogels was observed to be homogenous, suggesting an uniform and ordered material structure which was able to allow cells to adhere. Furthermore, cell phenotype on the surface of sample A and, to a lower extent, sample D, resembles to that of cells attached to a substrate. However, clear distinction could be made after 7 days of culture between ASCs phenotype in the presence of HA versus CS, since cells in samples C and D appear to be smaller and more rounded in shape than those in samples A and B, which display fibroblast-like phenotype.

**Figure 5:**
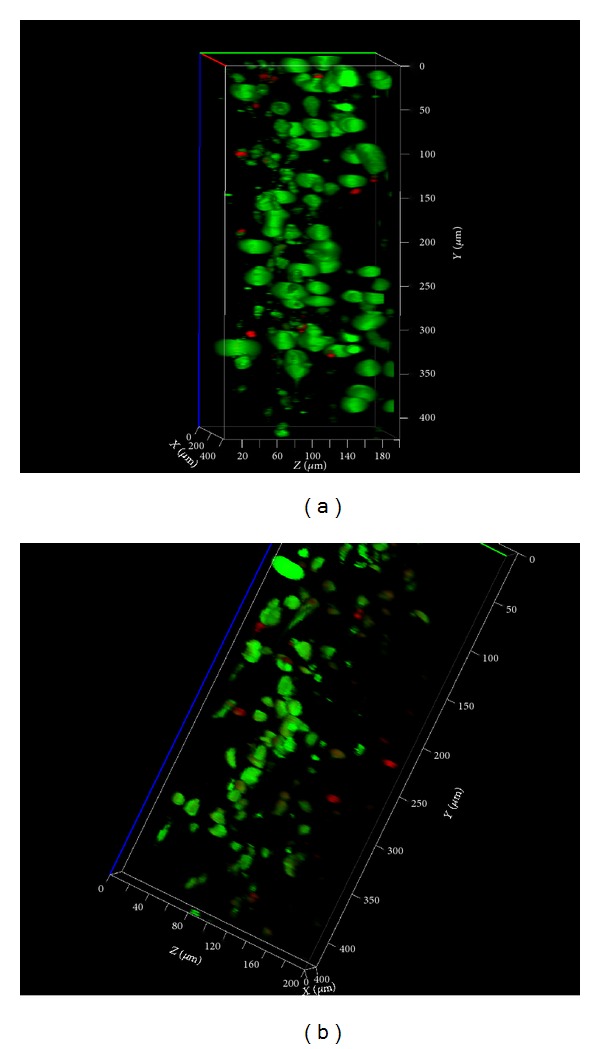
Three dimensional reconstructions of the z-stacks obtained by confocal microscopy in (a) ASCs-Coll-SS : HA 10% (sample A) and (b) ASCs-Coll-SS : CS 5% (sample D) Live/Dead labeled systems 7 days after seeding.

Cell distribution inside samples A and D was investigated at day 7 by laser scanning confocal microscopy. Three dimensional reconstructions of the scanned volumes (Figures 5(a) and 5(b)) revealed important differences in cell distribution and density in the presence of 10% HA (sample A) and 5% CS (sample D). Although ASCs were seeded at the same cellular density on the surfaces of these materials, a higher density was detected in sample A after 7 days of culture in contact with collagen, sericin, and 10% HA than in sample D. ASCs were able to populate more evenly the whole thickness of 200 *μ*m analyzed by confocal microscopy in sample A than in sample D, where all cells were distributed within 160–170 *μ*m. Additionally, ASCs detected in sample D appear to be smaller, as compared to the cells in sample A. 

The highly positive ratio between live (green-labeled) and dead (red-labeled) cells in both systems was confirmed once more through confocal microscopy (Figure 5).

#### 3.2.2. MTT Quantitative Evaluation of ASCS Viability and Proliferation Potential inside the Novel Coll-SS-Based Scaffolds

**Figure 6:**
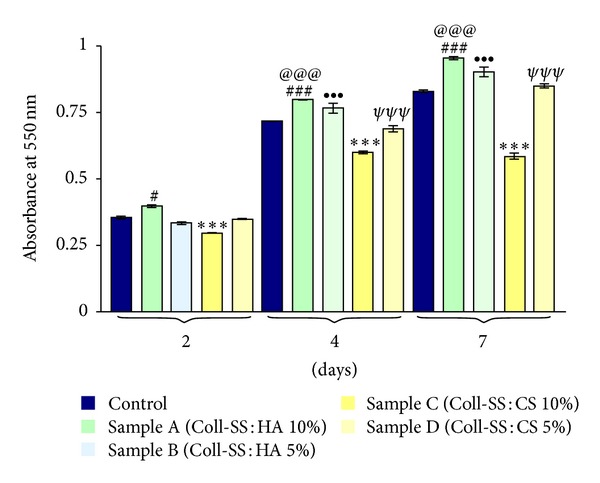
ASCs viability and proliferation profile evaluation after 2, 4, and 7 days of culture using spectrophotometric MTT assay ^#^ *P* < 0.05 (sample A versus control at 2 days); ^###^ *P* < 0.001 (sample A versus control at 4 and 7 days); ***P < 0.001 (sample C versus control at 2, 4, and 7 days); ^@@@^ *P* < 0.001 (sample A at 4 days versus sample A at 2 days and sample A at 7 days versus sample A at 4 days); ^•••^ *P* < 0.001 (sample B at 4 days versus sample B at 2 days and sample B at 7 days versus sample B at 4 days); ^*ψψψ*^ *P* < 0.001 (sample D at 4 days versus sample D at 2 days and sample D at 7 days versus Sample D at 4 days).

To validate the viability and the proliferation rate, MTT assay was employed as a more accurate approach. In this context, the Coll-SS scaffold as well as the samples A, B, C, and D were seeded with ASCs and subjected to MTT spectrophotometric assay at 2, 4, and 7 days of culture (Figure 6). 

Regarding the cellular viability, our results show that at 2, 4, and 7 days of culture the amount of metabolically active cells in sample A was found to be significantly increased (*P* < 0.05, *P* < 0.001, and *P* < 0.001) as compared to the control hydrogel. In contrast, during the entire experimental period ASCs cultured in sample C displayed a significant decreased viability profile (*P* < 0.01, *P* < 0.001, and *P* < 0.001). Samples B and D sustained cell viability during one week in a similar extent as the control hydrogel did, as no significant differences were registered between them. 

As shown in Figure 6, except sample C, all studied scaffolds allowed cellular proliferation. Although cells seeded in sample C did proliferate during the first 4 days of culture, between the 4th and the 7th day of experiment, the absorbance values remained constant. The control hydrogel, as well as samples A, B and D displayed a constant significantly increased (*P* < 0.001) proliferation profile, both at 4 and 7 days after seeding as compared to the previous time point. 

#### 3.2.3. Evaluation of LDH Release as Quantitative Determination of the Cytotoxic Potential of the Novel Coll-SS-Based Scaffolds

**Figure 7:**
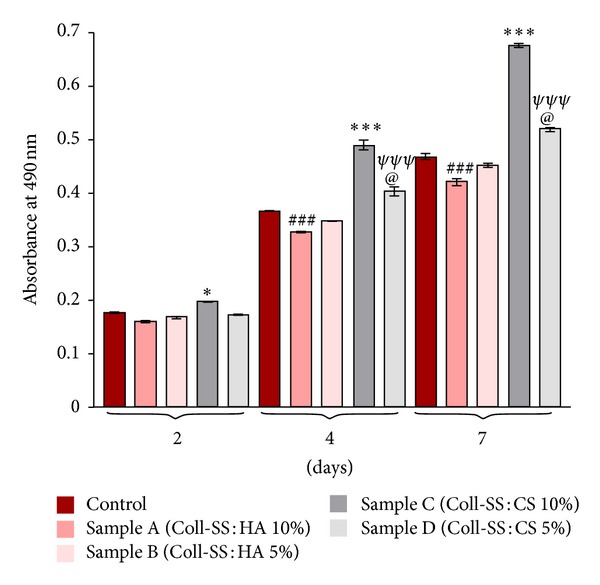
Collagen-based scaffolds cytotoxic potential evaluation by spectrophotometric LDH assay after 2, 4, and 7 days of culture (*P < 0.05 (sample C versus control at 2 days); ***P < 0.0001 (Sample C versus control at 4 and 7 days); ^###^ *P* < 0.001 (sample A versus control at 4 and 7 days); ^@^ *P* < 0.05 (Sample D versus Control at 4 and 7 days); ^*ψψψ*^ *P* < 0.001 (sample C versus sample D at 4 and 7 days)).

The cytotoxic potential of samples A, B, C, and D was evaluated by spectrophotometric quantification of the LDH enzyme release in the culture media by the embedded ASCS. A collagen-sericin hydrogel, unimproved with HA or CS, preseeded with the same number of ASCs, was used as reference (Figure 7).

At 2 days of culture only sample C displayed significant cytotoxic potential on ASCs as compared to control (*P* < 0.05). At 4 days after seeding, sample A showed a significant decreased cytotoxicity (*P* < 0.001) as compared to control, while at 7 days, almost the same significant decrease (*P* < 0.001) of approximately 10% was registered for this hydrogel, which proves a constant decreased ratio between this sample and the control during the experiment.

Although sample B displayed a constant ratio of cytotoxicity as compared to the control during the experiment, sample A showed significant lower values as compared to it. In addition, at 7 days of culture, the cytotoxic potential of sample A was found to be lower (*P* < 0.001) than that of sample B, showing that sample A could be a better candidate for further studies among the two HA enriched compositions.

Regarding samples C and D, starting with the 4th day of culture, both displayed a significant cytotoxic effect on ASCs (*P* < 0.001). The addition of 10% CS in the biomaterial composition increased the LDH levels with approximately 34% at 4 days after seeding and with approx. 45% after 7 days of culture. These increases are statistically significant (*P* < 0.0001) and suggest that sample C is not proper for further *in vitro *studies. In addition, the use of only 5% CS in the composition of the hydrogel also significantly decreased its cytotoxicity (*P* < 0.05). Thus, at 4 and 7 days of culture, the registered decreases were of approx. 12% and 17%, respectively, showing that sample D could be further used for *in vitro *studies.

Correlating the results obtained via quantitative and microscopy assays, we can generally conclude that the ratio between live and dead cells is strongly positive for all the studied bioconstructs, reflecting good biocompatibility of these materials. However, cells inside sample C were proved to have lower than 80% of viability, which makes it not eligible for further studies. Conversely, significant higher cell viability and lower cytotoxic potential were displayed by samples A and D, when compared to the other samples and to the control system. This observation suggests that not only the ratio between live and dead cells inside the scaffold, but also the material's formulation effect on cell viability is critical for cell behavior analysis in contact with novel materials proposed for tissue engineering.

## 4. Conclusions

The scaffolds prepared in this study show similar physical properties, having a porous structure with pore sizes between 20–150 *μ*m, high capacity of water absorption, and undenaturated triple helical structure of collagen. These properties make them promising candidates for cartilage tissue engineering. 

The results obtained using Live/Dead assay confirmed the quantitative determinations performed during MTT and LDH tests, suggesting that all samples are biocompatible, but the most equilibrated formulas in terms of cell behavior were collagen-sericin hydrogels improved with 10% HA and 5% CS (samples A and D). Furthermore, the scaffold's structure and pore distribution in both samples allow cell viability and proliferation, but sample A pore interconnectivity and composition favor ASCs distribution in deeper layers of the hydrogel than in sample D, simultaneous with maintaining the appropriate conditions for cell survival in the depth of the material.

MTT and LDH assays revealed that sample A exerts no cytotoxicity on ASCs and allows their proper proliferation, while regarding the CS compositions, sample D could be used for further studies of chondrogenesis but taking care of maintaining its cytotoxic potential at a level that does not interfere with cell proliferation or differentiation. Sample C was found to be cytotoxic and was excluded from further studies.

Since scaffold physical characterization revealed no significant differences between the samples, ASCs behavior in contact with each sample composition served as 3D system selection criteria for further *in vitro* chondrogenesis studies. Therefore, a combination approach including sample A and sample D properties could be a very promising solution for CTE studies.

## Figures and Tables

**Table 1:** Thermal properties of Coll-SS-based scaffolds, during the heating process.

Samples	Denaturation temperature (°C)	Melting temperature (°C)	Thermooxidation temperature (°C)
Control	58	219	310
Sample A	66	222	309
Sample B	63	216	310
Sample C	62	219	310
Sample D	56	219	310

*Experimental error: ±0.2°C.
