# Correction to “Biocompatibility Assessment of Novel Collagen‐Sericin Scaffolds Improved with Hyaluronic Acid and Chondroitin Sulfate for Cartilage Regeneration”

**DOI:** 10.1155/bmri/9828169

**Published:** 2026-06-22

S. Dinescu, B. Gălăţeanu, M. Albu, et al., “Biocompatibility Assessment of Novel Collagen‐Sericin Scaffolds Improved with Hyaluronic Acid and Chondroitin Sulfate for Cartilage Regeneration,” *BioMed Research International*, no. 2013 (2013). https://doi.org/10.1155/2013/598056.

In the article titled “Biocompatibility Assessment of Novel Collagen‐Sericin Scaffolds Improved with Hyaluronic Acid and Chondroitin Sulfate for Cartilage Regeneration”, there were multiple errors in Figures 2 and 4. These errors are shown and corrected below:

Figure 2

The SEM image of sample C in panel (d) was inadvertently used as an image of sample D in panel (e). This error occurred due to an oversight by the authors during figure assembly and should be corrected as follows:

**Figure 2:**
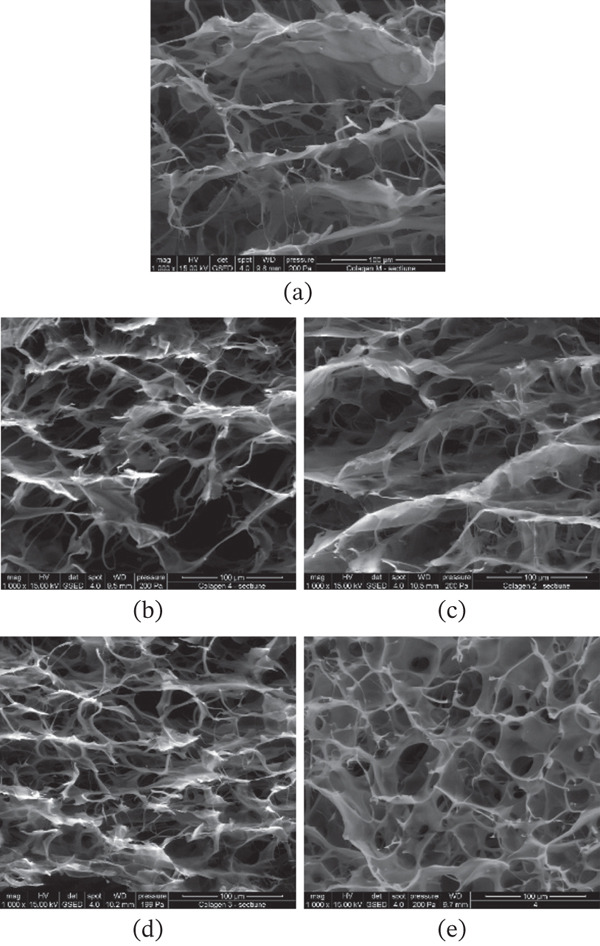
SEM images of (a) Coll‐SS, (b) Sample A, (c) Sample B, (d) Sample C, and (e) Sample D.

Figure 4

The image representing the fluorescent staining of the control sample after 4 days was inadvertently used to represent the staining of sample C after 7 days. This error occurred due to an oversight by the authors during figure assembly and should be corrected as follows:

**Figure 4:**
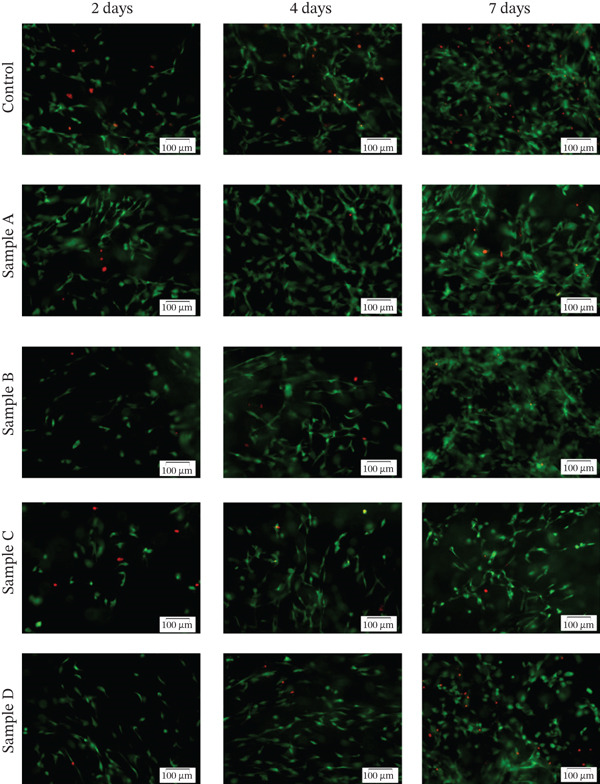
Fluorescence microscopy assessment of living (green‐labelled) and dead (red‐labelled) ASCs at 2, 4, and 7 days postseeding on Samples A, B, C, D, and the control.

We apologize for these errors.

